# A Prehospital Emergency Psychiatric Unit in an Ambulance Care Service from the Perspective of Prehospital Emergency Nurses: A Qualitative Study

**DOI:** 10.3390/healthcare10010050

**Published:** 2021-12-28

**Authors:** Lizbet Todorova, Anders Johansson, Bodil Ivarsson

**Affiliations:** 1 Office of Medical Services, University Trust, Region Skåne, 221 85 Lund, Sweden; Lizbet.Todorova@skane.se (L.T.); anders.johansson@med.lu.se (A.J.); 2 Clinical Sciences, Faculty of Medicine, Lund University, 221 85 Lund, Sweden

**Keywords:** ambulance services, competence, content analysis, interprofessional, psychiatric care, mental illness, non-conveyance, prehospital emergency care, specialist nurse, team

## Abstract

The prevalence of mental illness is steadily increasing, and ambulance teams frequently attend cases with suspected mental illness. A pilot project, Psychiatric Emergency Response Team (PAP), was carried out in which a prehospital emergency nurse (PEN) was accompanied by a psychiatric specialist nurse in the assessment of individuals with mental illness. The aim of the present study was to evaluate a prehospital emergency psychiatric unit from the perspective of PENs. A qualitative method using content analysis was applied. Seven senior PENs who had worked for 1 year in a prehospital psychiatric ambulance unit were interviewed individually. The analysis resulted in one main theme, “Transition from limited care and insufficient competence to improved and adequate care for psychiatric patients in ambulance care”. This emerged from six subcategories: *inter-professional development, access to patient records, the* *ambulance vehicle,* *non-conveyed patients, cooperation with the police and meetings with patients and next of kin*. In conclusion, these results suggest that in ambulance care in general, there is a lack of knowledge and skills about mental illnesses and initial care options. The PAP concept opened new avenues for the care of patients with mental illness, which the PENs described very positively as being helpful and valuable.

## 1. Introduction

Mental illness is a fast-growing health problem and can take on many forms, including changes in emotion, thinking, behavior, or a combination of these. Environmental factors that can affect mental health are associated with distress and/or problems affecting social, work, or family events [1]. Individuals suffering from serious mental illness are at higher risk of experiencing a range of chronic physical conditions, whereas individuals with chronic physical health conditions can experience depression and anxiety more often than the general population [2]. Alcohol and drug use disorders, depression, schizophrenia, and bipolar disorder are among the ten leading causes of disability worldwide [3]. Co-existing mental and physical conditions can diminish quality of life and lead to longer illness duration and worse health outcomes. Such conditions could be experienced severely and require immediate contact and help from emergency medical services. A previous study has shown that mental health patients use emergency medical services at a higher level than the rest of the general population [4].

Emergency calls to alarm operators regarding mental crises have increased and account for 40% of all incoming emergency calls, and a large proportion of these callers are also identified as recurrent users of ambulance care [5]. Patients with various mental illnesses often pose difficult challenges for alarm operators and paramedics in evaluating, triaging, treating and referring [6,7]. Despite the existence of various treatments, many individuals with a mental disorder do not have access to effective treatments [8]. Assessing cases of mental illness is becoming a significant part of the ambulance nurse and paramedic workload, and there is a need for education and training specific to mental illness [8], not least of all when a significant proportion of patients with psychiatric symptoms are assessed and left at home by the ambulance team with self-care advice or referred to a care provider other than those accompanying an ambulance [9].

Prehospital emergency specialist nurses (PENs; a registered nurse with 1 year of additional training in emergency care, also referred to as an ambulance nurse) need to have the appropriate knowledge and competence to ensure that both somatic and psychiatric illnesses are adequately recognized and treated. In some parts of Sweden, an ambulance unit with ambulance personnel and a psychiatric specialist nurse has been described to provide improved care for individuals with mental illness and reduce unnecessary transport to hospitals, the allocation of emergency ambulances, and the stigmatization of mental illness [10,11,12].

Lately, a similar first-line ambulance response, a prehospital emergency psychiatric unit (PAP), has been formed as a pilot project in the southeast part of Sweden, in which PENs are accompanied by a specialist trained psychiatric nurse (PN) to assess cases with mental illness. Evaluation of the PENs’ competencies prior to commencing the project revealed that the PENs had limited skills and competence, and that interprofessional collaboration with the PN was expected to increase the PENs’ general knowledge about various psychiatric illnesses, treatment options, and interfacility knowledge for continued care [13]. As the development of competence is strongly affected by the ability and possibility to reflect on practice on a professional and personal level, particularly in cooperation with colleagues within ambulance care [14], the present study aimed to evaluate a prehospital emergency psychiatric unit from the perspective of prehospital emergency nurses.

## 2. Methods

### 2.1. Design

A qualitative, descriptive, and retrospective design utilizing conventional content analysis was used [15] and reported according to the COREQ checklist [16].

### 2.2. Study Setting and Population

In the autumn of 2019, a project with a complementary PAP was started in a part of the County of Skåne with 1.3 million inhabitants and approximately 160,000 ambulance assignments annually. The PAP was operating from 3 p.m. to 1 a.m. every day. The team consisted of two registered nurses (RNs) with an additional 1 year of higher education, one specialized in prehospital emergency care and one in psychiatric care. The team vehicle resembled an ordinary ambulance and was equipped with blue lights and sirens, a computer for mobile access to medical records, and medication supplemented with basic sedatives, sleep drugs, and antipsychotic drugs. Data were collected after the first year of the project, which ran between October 2019 and November 2020. The study included all seven senior ambulance nurses (2 females and 5 males) involved in the project, all with ≥5 years of professional nursing experience.

### 2.3. Ethics Approval and Consent to Participate

Ethics approval was obtained from the Swedish Ethical Review Authority (Dnr: 2019-04040). Participation in the study was voluntary and the respondents were informed that confidentiality was guaranteed and that they could withdraw participation at any time without explanation. All data were stored securely, and access was permitted only to the research team.

### 2.4. Data Collection

Inspired by Todorova et al. [13], semi-structured interviews asked the following questions of the respondents: “Regarding this year with PAP, what did it mean to you?” “Can you tell me about your experiences caring for patients with mental illnesses in PAP?” They were complemented by follow-up questions for clarification: “Can you tell me more?” and “In what way?” One pilot interview was conducted to test the appropriateness of the questions. No changes were needed after the test interview. All individual, face-to-face interviews lasted between 24 and 46 min, were carried out by author B.I. (an RN and PhD with extensive experience of qualitative interviewing) in a dialogue form, digitally recorded, and then transcribed. Author B.I. had also conducted the first interview round with the same informants one year earlier [13].

### 2.5. Data Analysis

The verbatim transcripts were independently read several times and coded by two nurse researchers with qualitative research experience using a conventional content analysis according to the procedure proposed by Hsieh and Shannon [15]. Conventional content analyses are used when there is no starting point other than the data itself, also called a bottom-up approach. The data analysis was carried out using Microsoft Word’s Tools [17]. During the stepwise analysis, meaningful text units were identified that corresponded to the aim of the study. These units were copied from the text, and each meaningful object received a code that highlighted what the text contained. The code words were then organized into subcategories, categories, and a main theme. Throughout the analytical process, all authors repeatedly discussed the categories until reaching an agreement. Quotes from the respondents’ (R1–R7) interviews were identified and used to illustrate and underpin each subcategory.

## 3. Results

**Figure 1:**
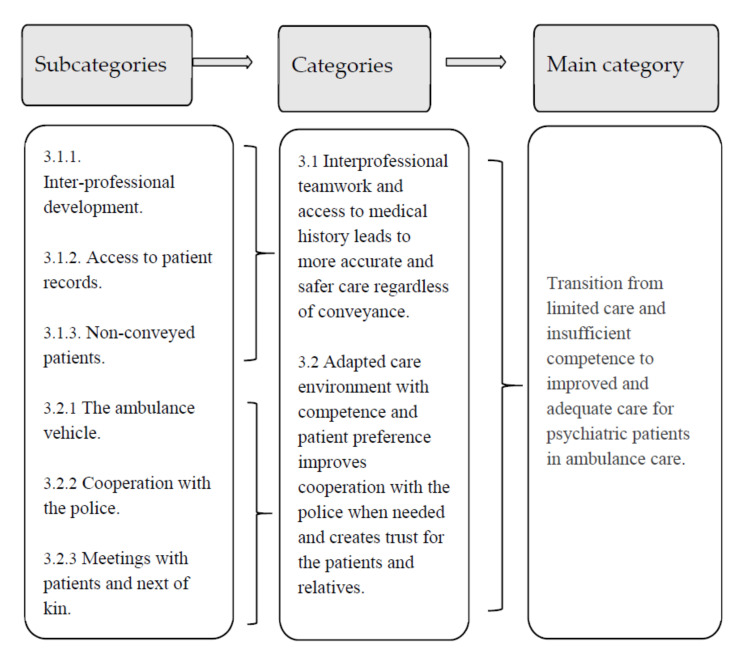
Subcategories, categories, and main theme based on the prehospital emergency nurses’ overall perception of their working experiences in a psychiatric emergency response team.

The two categories and their subcategories together led to the overall theme, “Transition from limited care and insufficient competence to improved and adequate care for psychiatric patients in ambulance care” (Figure 1), which encompassed the PENs’ descriptions of how participation in a prehospital emergency psychiatric unit increased their competencies’ when assessing patients with mental illness.

### 3.1. Interprofessional Teamwork and Access to Medical History Leads to More Accurate and Safer Care Regardless of Conveyance

#### 3.1.1. Inter-Professional Development

The PENs described that working in teams with a PN meant that they were able to confirm their previous knowledge acquired through a mandatory prehospital psychiatric care program. The PENs also considered that they continuously learned new perspectives by seeing and listening to how the PN obtained a medical history and met and treated patients with mental illness. Even during patient-free periods, an exchange of knowledge took place; the PENs described that the PN was interested in learning more about ordinary ambulance care, including somatic diseases, which meant that they experienced mutual learning. This mutual learning also included experiences of personal safety in this context; the PENs expressed that they had a great responsibility to teach the PN personal safety strategies in both the public environment and patients’ homes.

*“My understanding of the psychiatry context and the psychiatry nurse’s challenges has increased… and their understanding of the somatic part of the ambulance care has improved. This together has led to better preliminary diagnoses and triage among the psychiatric patients. These patients get a double assessment that they do not get anywhere else… we together become more like one unit. And for me personally, it will take some time to become even better in the encounter with these patients, but I already feel a positive difference after 1 year of participation in this project”.*

(R4)

#### 3.1.2. Access to Patient Records

In Swedish ambulance care, it is not common to have access to patients’ medical records. The project made this possible. The PENs described this access as very important—that it was possible for the team to see on the way to the patients if violence had occurred in connection with these patients and, if so, the team had the opportunity to request police support for patient safety and the security for the team. Furthermore, the team could discuss and prepare the patient’s care by reading whether the patient had a personal treatment strategy, what medications they may have been treated with, what symptoms they had, and for what reasons the patients previously had contacted different health care providers. Once on-site, the patients were informed that the team had read their medical records and that the patients did not have to repeat their medical history, which the team felt the patients appreciated. Regardless of whether the patients were left at home or handed over to another care provider, the PN could make notes about what they had done and/or send messages to a care provider with which the patients may already have had contact.

*“Access to medical record systems is fantastic for further treatment opportunities… to be able to be prepared before coming to the patient. Many of these patients do not have the strength or find it difficult to talk about certain things. Every time a regular ambulance unit handles a similar situation, you do not really know what the conversation should be about and sometimes you become unsure whether the patients tell the whole story of their illnesses”.*

(R1)

#### 3.1.3. Non-Conveyed Patients

The informants believed that the team left patients at home or referred them to another care facility the next day to a greater extent than a regular ambulance team. They reasoned that it was because they could read the medical records and, thus, allow patients to be more involved in their care. In addition, the encounter and treatment options were facilitated by the PN being equipped with an expanded drug arsenal that consisted of anti-anxiety, sedative, or sleep-inducing drugs that could be given by delegation or in contact with on-call psychiatrists. The team felt that the patients appreciated not being transported to a psychiatric emergency department but were advised, instead, to visit their regular care provider the next day. An example given was “acute crisis response”, which was described as a condition in which the patient was not really ill but something unexpected had happened for which conversational support and temporary medication could help in a better way than being transported to a psychiatric emergency department.

*“It [expansion of drugs] means that we can treat more patients at home, that you do not always have to go to a psychiatric emergency… from the patient’s perspective it emerges that you [the patient] can get help at home, you can probably manage the situation until the next day and visit your own physician… you end up at the right level of care from the beginning. So, I think it has made a big difference”.*

(R5)

### 3.2. Adapted Care Environment Improves Cooperation with the Police and Creates Trust for Patients and Relatives

#### 3.2.1. The Ambulance Vehicle

A special psychiatric ambulance unit was used in the project and the PENs felt secure because it was, to a great extent, equipped like a regular ambulance. In many cases, it is difficult to assess a case based on a telephone call regarding whether the medical need is of a somatic or psychiatric nature. However, the present ambulance unit had chairs instead of a stretcher, which the informants described as an environment inviting more open dialogue and communication, which they presumed the patients appreciated. Nothing on the psychiatric ambulance revealed that the unit was intended for psychiatry missions, which was appreciated by most members of the team who believe that mental illness is still stigmatized in society.

*“Wow, is there no stretcher here?”… The patients seem to like this… it becomes more like a small chat room and the atmosphere does not become so “unhealthy”, though we can sit on chairs instead of the patient lying on a stretcher”.*

(R6)

#### 3.2.2. Cooperation with the Police

The informants pointed out that an encounter with patients with mental illness is not equivalent to violence, but they are prepared before the assignment. The informants described that their cooperation with the police force had increased in a positive way during the pilot project and they could quickly and directly contact the police force if needed.

PENs also described that the PAP project has improved care in compulsory care, where the purpose is partly to protect the patients from themselves and partly to protect society from the patient. In regards to patients with known psychiatric diseases, the PN could contact a psychiatry physician by telephone about a compulsory care situation, and this procedure also made it easier to obtain help from the police force if it was deemed necessary. The fact that collaboration has increased and improved was partly perceived due to information about the project and the increased competence in the project (e.g., the participation with the PN).

*“We have been treated very positively by the police force. They can now get a short report about the patient and they know that there is a specialist trained nurse in the ambulance who knows what it is about. It is not just someone [the psychiatric nurse] who has made a hasty decision that this patient should enter the hospital, no, there is, in fact, a formal theoretical knowledge behind this decision”.*

(R7)

#### 3.2.3. Meetings with Patients and Next of Kin

The informants’ perceptions were that both patients and their next of kin were generally satisfied and felt secure in receiving assistance from the project team. However, there were patients who did not experience that they had symptoms in the context of psychiatry and, therefore, were not as enthusiastic when a psychiatric ambulance arrived. On the other hand, if the patients had previously been in contact with the PAP team, the informants observed that some patients waited to call the dispatch center until they knew that the psychiatric ambulance was on duty. The informants agreed that this patient group had complex needs regarding their illness and social situation, and that they should be treated with respect even though the informants sometimes felt powerless in their attempts to help the patients.

*“The patients who call the dispatch center many times, we know them in the ambulance service. We will never get rid of it, only the people change. We just have to learn to deal with them…I know and understand why they exist and how much they affect the rest of the system. Because these patients take a lot of attention”.*

(R2)

## 4. Discussion

Our results suggest that the PENs’ experience of the PAP project opened up new dimensions of care for patients with mental illnesses. The interaction between the PENs and PNs increased inter-professional development. Based on our results here and previously [13], the interprofessional collaboration between the ambulance service and psychiatric department appears to be crucial when an individual is suffering a mental illness and in need of emergency psychiatric care. During the past year, PENs have experienced that, overall, they felt much more competent and safer and can now provide professional and dignified treatment. The PENs also felt that both patients and their next of kin were generally satisfied and felt secure in receiving assistance from the project team.

In the present study, the participation of PNs was perceived as creating increased confidence for the patient; for example, the PN was thought to make more reliable assessments of the patients. Previously, when assessing patients that repeatedly need emergency care, the ambulance nurses have described a feeling of resignation due to a lack of knowledge about appropriate care and alternatives for further levels of care [13]. Mutual and correct initial assessment aided the PENs in identifying patients with complex mental illnesses. Patients with symptoms of high complexity/severity, commonly identified as recurrent patients in the ambulance, preferred the PAP unit when in need of emergency care. 

A caring environment that is calm and confident is essential for the ambulance nurse to prepare and create conditions that facilitate nursing care [18]. The PENs’ perception was that the psychiatric ambulance should be anonymous, which patients in a previous study also noted with reference to the stigmatization of mental illness in society [12]. This is an important observation, as an improved assessment approach and tools in ambulance care may lead to the detection of early signs and symptoms of psychiatric illness at the first visit and possibly prevent the onset or diagnostic delay of psychiatric disorders. In addition, it emerged that access to patient records in combination with an assessment of the patient’s medical condition facilitated the collaboration between the PAP team and the patients’ regular care providers. This made it possible for more patients to stay at home; the patients avoid waiting times in emergency rooms and possible admissions to the ward if they are referred to the correct level of care [19]. Furthermore, in cases of an acute crisis response, a PEN could alleviate the patient’s sudden suffering so they can safely stay at home until they visit their own physician. Our informants experienced that the collaboration with the PNs in the assessment situation generated increased patient satisfaction. 

It was previously confirmed that to ensure appropriate patient assessment, different nurses must meet the competency requirements for assessing patients with mental illness [20]. These competency-related demands often include situation awareness, history gathering, communication, and procedural skills [21]. A previous study established that situational awareness in ambulance care can lead to improved results [22]. Situational awareness includes recognition (i.e., creating a perception and picture of the situation), interpretation to achieve an understanding of the situation, and predicting and planning for an event [22]. The present study highlighted the team’s opportunities to prepare for the prehospital assignments that were psychiatric in nature by obtaining information from the patients’ medical records, thereby making use of situational awareness. This approach can also be useful when facing threatening situations. Moreover, the PENs highlighted personal safety and the importance of both members of the team having knowledge and skills regarding threats and violence, which is relatively common in prehospital psychiatric care meetings with patients and relatives. The most common causes of threatening behavior and chaotic situations are the effects of alcohol, drugs, and mental illness [23].

Several psychiatric disorders are considered chronic conditions in which an individual has recurrent outcomes, such as hospitalization, symptomatic relapse, or self-harm behavior [2], which often lead to the recurrent need for an ambulance. In our study, the PAP team described a feeling of powerlessness when attending frequent callers. Analyses of recurrent events in emergency psychiatric research are sparse, but it can generate an opportunity to address research questions aimed at understanding the longitudinal course of a chronic condition in relation to mental illness [2]. Such approaches may provide novel insights into risk factors or interventions for psychiatric illness and perhaps improved outcomes for individuals who are referred as recurrent/frequent callers to the emergency dispatch center and in need of ambulance care. For example, it has previously been shown that collaboration with primary care and social services and individual care plans is able to help many frequent callers reduce their contacts with dispatch centers [24]. The PAP team having access to individual care plans can be a way to help many people and, perhaps, make the inevitable meetings confident but short. Another aspect is the collaboration with the police. Experiences from a similar psychiatric emergency response team in Sweden [10] showed that patients with psychiatric disorders received high-quality prehospital assessments, which reduced the workload of the police and ambulance services. Thus, improved knowledge of the interprofessional organization and collaborative efforts with cross-sectoral coordination to treat are factors that may contribute to reducing the stigmatization of psychiatric illnesses.

To ensure that patient assessments are adequate and appropriate, ambulance nurses’ mental health competencies must correspond with the requirements of their profession. Earlier results identified three key areas of competency for assessing patients with mental illness in emergency care: adequate theoretical and practical knowledge, extended communication skills, and a respectful attitude [20]. This result is in line with Edmund et al., who concluded that education and training, organizational factors, and clinical decision-making are important in ambulance care to ensure an appropriate response to the needs of people experiencing mental health issues [8]. This study suggests that the support of PNs in ambulance care creates broader and improved perspectives for PENs according to the conditions for patient participation and, therefore, increases patient satisfaction in ambulance care. Our results show that the PNs strengthened the assessment situation through their extended knowledge and skills but also via access to the medical record. Most importantly, we think this generates more accurate assessments and confident care for patients who suffer from mental illness in situations where the patients exhibit symptoms that are both mental and somatic in nature. Whether the present concept will constitute best practice within ambulance care for mentally vulnerable individuals and relatives awaits further studies on patient perceptions of the given care and outcome.

A variety of acute prehospital models open the way for further investigation regarding resources and economic aspects. For example, in the UK, Crisis Resolution Teams (CRTs) that provide short-term, intensive home treatment for people experiencing an acute mental crisis are widespread but has also entailed risks of discontinuity in care [25]. Therefore, different evaluations of the mentioned models may be required to fully understand the effects of these organizational approaches.

### Study Limitations

A small group of informants is normally considered to generate low transferability. In the present study, all seven informants employed during the whole project were included. Although this is a limited number of informants, all of them have had more than 13 years of working experience, which we believe adds a deeper and reflective description of their experience before, during, and after working 1 year with the PAP unit. Length of working experience in the ambulance services is shown to be associated with higher professional competence [26]. We consider that our methodical process of analysis increases the study credibility because the coherency of interpretations was discussed and revised by all authors. We also believe that the informant answers in the interviews are genuine and of high importance to the informants. Because of the study’s qualitative nature, the aim was not to generalize the findings to apply to all acute mental care outside hospitals. However, it is reasonable to assume that the results can show a part of the reality of PENs’ experiences in connection to mental illness in ambulance care and give a better understanding of the prehospital interaction between different actors.

## 5. Conclusions

The PAP concept opened new avenues of care for patients with mental illness, which the PENs described very positively. What was described as developing for PENs was the increased communication skills and specific knowledge about various mental illnesses. After 1 year of working with the PAP unit, their overall perception included feeling much more competent, safe, and able to give more professional and dignified treatment. Their newly acquired knowledge increased their ability to interpret and understand the need for care when assessing patients with mental illness, which was considered to result in improved prediction and planning for an appropriate level of care for these individuals. Hopefully, in the future, it will contribute to equating mental and medical disorders and counteract the stigmatization of psychiatric disorders.

## Data Availability

The data presented in this study is available on request from the corresponding author.
